# Construction of Discrete Element Constitutive Relationship and Simulation of Fracture Performance of Quasi-Brittle Materials

**DOI:** 10.3390/ma15051964

**Published:** 2022-03-04

**Authors:** Ran Zhu, Hongbo Gao, Yijian Zhan, Ze-Xiang Wu

**Affiliations:** 1 Shanghai Construction Group Co., Ltd., Shanghai 200080, China; zhanyj@scg.cn; 2 Shanghai Engineering Research Center of Mega Structure High Performance Concrete, Shanghai 201114, China; 3 College of Civil Engineering and Architecture, Hainan University, Haikou 570228, China; 4 College of Civil Engineering and Architecture, Wenzhou University, Wenzhou 325000, China; zexiang.wu@wzu.edu.cn

**Keywords:** discrete element method, quasi-brittle material, constitutive model, mortar, fracture behavior, acoustic emission

## Abstract

In order to solve the problem that the built-in parallel bond model in the discrete element software cannot adequately simulate the post-peak fracture behavior of quasi-brittle materials, a linear cohesive model was established. First, two particles are used to simulate the interface constitutive behavior in different modes. The results show that the new model can better simulate the behavior of Mode-I fracture, Mode-II fracture, and Mixed-mode fracture. Then, the influence of micro-parameters on the newly constructed constitutive model is analyzed, which provides a basis for the determination of micro-parameter values. Finally, the proposed softening model is applied to a three-point bending test of mortar, and the fracture behavior obtained is compared to the acoustic emission results. The simulation results also show that the constitutive model we built can be used to simulate the fracture behavior of quasi-brittle materials such as mortar and concrete.

## 1. Introduction

Quasi-brittle materials such as concrete, mortar, rock, or wood are widely used in various engineering fields [1]. For quasi-brittle materials, fracture is a common phenomenon that affects structural safety and durability [2,3]. The failure cracking mechanism of these materials is usually complex (microcracks, crack bridging, crack rest, etc.), characterized by the formation of a large microcrack fracture process zone (FPZ) before the main crack. Due to the damage development in the process zone, the quasi-brittle materials have some special fracture characteristics, such as R curve and size effect [4]. The stress–strain relationship of quasi-brittle materials exhibits nonlinear characteristics before reaching the peak value, and a ‘strain softening’ phenomenon after the peak value. Therefore, research on the properties of quasi-brittle materials should start with their mesostructure, focus on their heterogeneous composition, and appropriately investigate the damage and fracture mechanism of quasi-brittle materials according to their meso-mechanical properties.

The numerical simulation method can effectively solve the limitations of fracture tests and crack measurements, which is beneficial to systematically estimate the related performance of the structure, and save the resources required for experimental research. Simulation of nonlinear problems in quasi-brittle materials, especially damage and degradation, is one of the popular research tasks in current civil engineering [5]. However, due to the existence of the fracture process zone in quasi-brittle materials, the classical linear elastic fracture mechanics (LEFM) theory cannot be directly applied to quasi-brittle materials [6], and the propagation of cracks in quasi-brittle materials is a discrete problem, which should be studied locally. Compared to the finite element method (FEM), the discrete element method (DEM) focuses on meso-mechanical problems, which can be controlled at the particle level, and can effectively solve discontinuous problems mentioned previously. The discrete element method was originally developed by Cundall [7] to solve rock mechanics problems. Although some studies [8,9,10] have successfully used the DEM to simulate the fracture behavior of concrete, how to choose a suitable contact model remains a key issue of the DEM while simulating the fracture behavior of quasi-brittle materials.

To perform DEM simulations, several computer codes have been implemented, such as PFC (Particle Flow Code), UDEC (Universal Distinct Element Code), LIGGGHTS (LAMMPS Improved for General Granular and Granular Heat Transfer Simulations), etc., among which PFC has been extensively used. In PFC, the parallel bond model [11,12,13] is one of the most frequently used built-in contact models. The previous studies usually focus on some specific mechanical behaviors of quasi-brittle materials and focus only on the mechanical response at peak load, but pay little attention to the post-peak behavior. Zhou et al. [11] successfully simulated the pre-peak rupture mechanism of concrete using a parallel bond model. Murali et al. [14] have studied the strength and size effects of tensile concrete using the parallel bond model, which can be used to describe the pre-peak fracture behavior of concrete and its peak value. However, Jiang et al. [15] pointed out that the parallel bond model cannot satisfy both compressive and tensile behavior when using the same microscopic parameters. The results of Diederichs [16] showed that the tensile strength of rocks was remarkably overestimated when the parameters were calibrated using compression tests. Prior to Diederichs, Potyondy [17] and Schopfer [18] obtained similar results in their simulations of brittle rocks, because the macroscopic tensile and compressive strength increased simultaneously with the increase in the bond tensile strength in the microscopic model [13]. In fact, compressive strength is usually one order of magnitude greater than tensile strength in rock, concrete, and other similar quasi-brittle materials. As a result, the parallel bond model can no longer be used to simulate quasi-brittle materials with low tensile/compressive strength ratios, such as rock and concrete.

Nguyen et al. [19] employed the DEM approach to simulate the three-point bending test using notched soft rock beams, and found that the parallel bond model could match the load–crack opening displacement (P-CMOD) curve, but could not capture its softening behavior. Therefore, Nguyen et al. [19] established an improved DEM damage–plastic cohesion model and verified its reliability. Ma and Huang [20] extended the normal equation of the built-in parallel bond model with a softening segment. The simulation results showed that the softening coefficient could reproduce the tension–compression ratio of quasi-brittle materials. Sinaie et al. [21] successfully developed a cohesive force model to simulate the behavior of concrete under cyclic loading, where the microscopic parameters of the material were calibrated only by a monotonic stress–strain test.

The previous studies of DEM simulation mainly focus on geotechnical materials, while the study on mortar, concrete, and other quasi-brittle materials is relatively inadequate, particularly with regards to fracture behavior. Due to of the influence of coarse aggregate inclusion and interface transition zone (ITZ), the fracture behavior of concrete is complex. It is easy to determine the basic fracture characteristics of mortar, as an important bonding material in concrete, through the study and simulation of its fracture behavior, because of the elimination of inclusion effect [22]. Therefore, in this study, the limitation of the parallel bond model for simulating the three-point bending fracture behavior of notched mortar beams is verified first, and then a linear cohesion model for the DEM to simulate the post-peak fracture behavior of mortar is developed.

## 2. Simulation of Fracture Behavior of Three-Point Bending Beams by Parallel Bond Model

**Figure 1:**
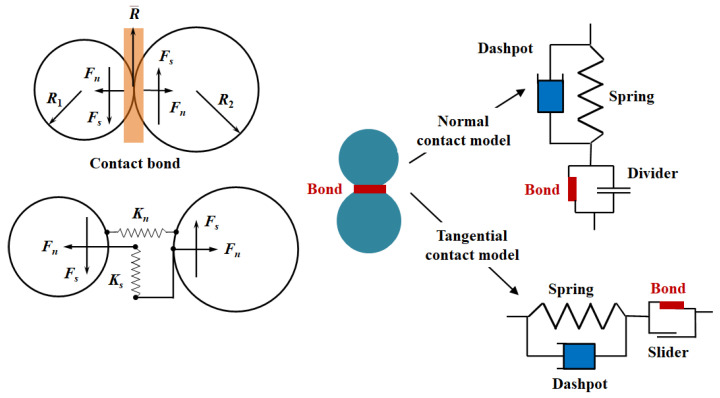
Mechanical behavior of parallel bond model based on [23].

The parallel bond model is a built-in constitutive model commonly used in the discrete element software PFC. This model consists of a linear sub-model and a sub-model for the bonding behavior (Figure 1). A two-dimensional parallel bond model shows that the two particles are cemented by a rectangular material with a certain strength, and can resist tension and torque. When bonds exist, the force on the model is mainly carried by the bond material. When bond failure occurs, the model degenerates into a linear contact model.

**Figure 2:**
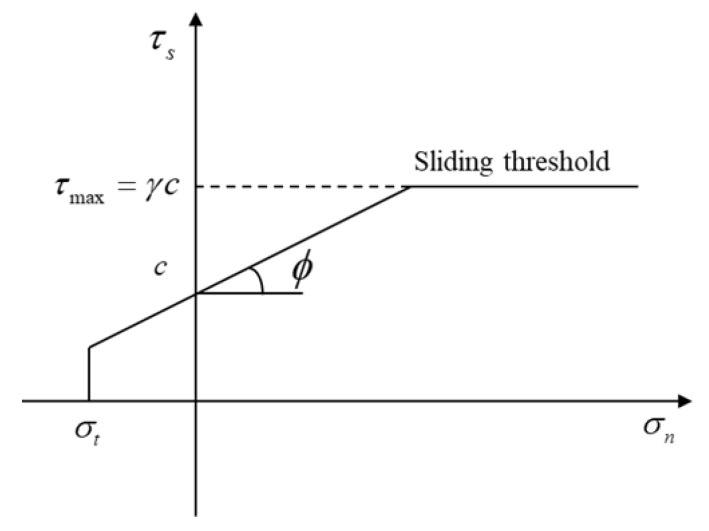
Strength curve of parallel bond model.

Figure 2 shows the constitutive relation of the parallel bond model, which reflects the relation between force and failure threshold. When the tensile stress between particles is greater than the tensile strength, the parallel bond exhibits tensile failure; when the shear stress between particles is greater than the shear strength, the parallel bond indicates shear failure.

**Figure 3:**
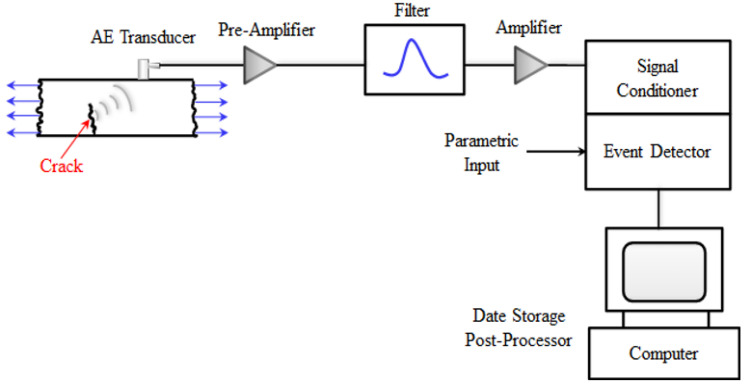

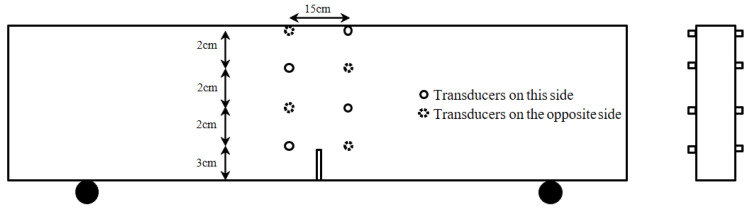
Schematics of AE instrumentation and positions of AE transducers.

To see if the parallel bond model is adequate for modeling the fracture behavior of quasi-brittle materials, it was put to the test. The 2D notched mortar beam (100 mm × 100 mm × 400 mm, the mixture shown in Table 1) was tested by three-point bending load. In the mortar, ordinary Portland cement (CEM I 52.5N) was used. The compressive strength was 48 MPa and 59 MPa on the 7th and 28th days, respectively, and the fineness modulus of natural sand was 2.16. The acoustic emission (AE) technique was also used to study the fracture process in this experiment. The acoustic emission transducers were attached to the opposite sides of the specimen through a coupling agent, organized to reduce inaccuracy in finding the AE occurrences around the projected position of the fracture process zone (FPZ). Thus, the sensor forms a parallelogram grid position on one side of the mortar specimen (60 mm × 150 mm), as shown in Figure 3.

According to previous research [24], the mechanical properties of the simulated specimen will be affected by the particle size when the particle size is larger than 1/80 times of the specimen size. Therefore, the range of particle radius, particle density, and local damping coefficient are set to be 0.5 mm ~ 0.75 mm, 2100 kg/m^3^, and 0.7 [25], respectively. The microscopic parameters are determined by the compressive strength of the 40 mm × 40 mm × 40 mm specimen and the flexural strength of the 40 mm × 40 mm × 160 mm specimen. In most cases, the calibration process is performed using the so-called “trial-and-error” method based on a standard test, although this method is time-intensive and results in a non-unique combination of micro-parameters. In this paper, a Levenberg–Marquardt (LM) algorithm-based calibration scheme is adopted; more detailed information can be found in the author’s previous work [26]. Many micro-parameters have to be determined for DEM modeling, and it is not trivial to obtain the values of these parameters. Nevertheless, the initial values of normal stiffness and shear stiffness for calibration can generally be calculated from engineering properties such as Young’s modulus and Poisson’s ratio using Equations (1) and (2) [27].

(1) $$k_{n}=\frac{E}{\sqrt{3}\left(1-v\right)}$$

(2) $$k_{s}=\frac{E\left(1-3v\right)}{\sqrt{3}\left(1-v^{2}\right)}$$

where E is the Young’s modulus and v is the Poisson’s ratio.

The calibrated microscopic parameters are listed in Table 2.

**Figure 4:**
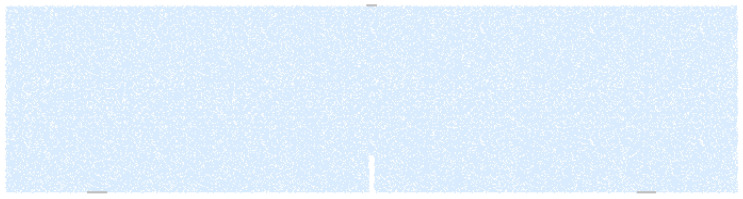
Mortar specimen shape and boundary condition.

**Figure 5:**
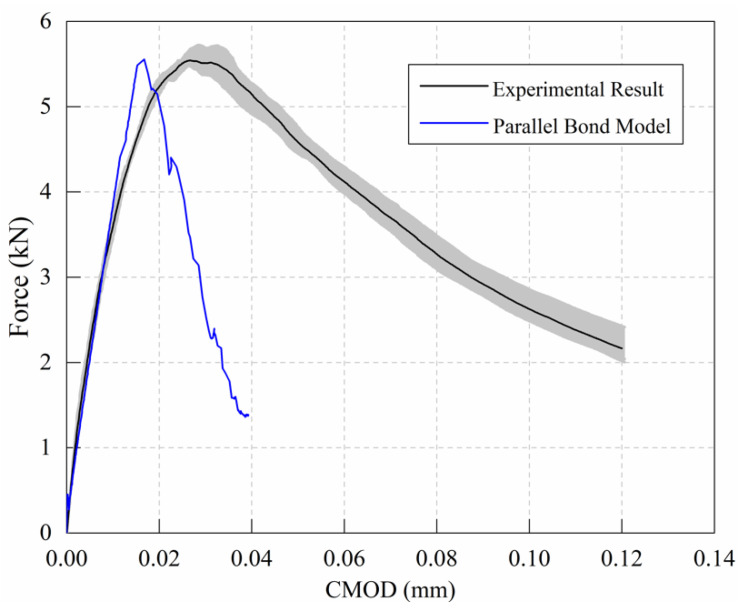
Comparison of parallel bond model results and test results.

The microscopic parameters obtained above were used to simulate the failure behavior of a notched three-point bending beam with a size of 100 mm × 100 mm × 400 mm. As shown in Figure 4, the simulated specimen was created by generating 80,821 particles within a rectangular specimen area. Taking the computation time into account, and ensuring that the specimen maintains quasi-static equilibrium during the test, based on previous studies [25,28,29,30], a constant vertical velocity of 0.002 m/s was applied at the top of middle span [31]. Figure 5 depicts the results obtained using the built-in parallel bond model and the experimental results.

The simulation results using the built-in parallel bond model agree well with the experimental results in the linear elastic ascending stage, according to the load–crack opening displacement curve in Figure 5. The simulation result is still in the linear growth stage, while the experimental result enters the nonlinear stage; therefore, the simulation and test results diverge gradually. When the loading reaches the post-peak stage, the parallel bond model degenerates into a linear model and no longer bears tensile load. This results in a rapidly descending curve, indicating a significant brittle fracture phenomenon. As a result, while the parallel bond model can simulate the peak value that corresponds to the test results, it can hardly replicate the nonlinear ascending stage and post-peak softening stage during the test process. Hence, the fracture behavior of quasi-brittle materials cannot be simulated by the built-in model.

## 3. Establishment of a Linear Cohesion Model

**Figure 6:**
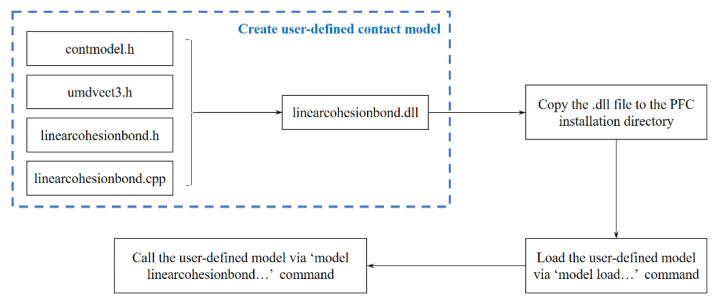
Flow chart of user-defined contact model implementation.

Fortunately, users are allowed to define their own contact models with PFC. The specific approach requires model coding in C++ language and compiling into a “.DLL” file, which is later called by PFC’s command flow. This process is illustrated in Figure 6 and the detailed information can be found in the official documentation of PFC [32]. The user-defined contact model is relatively independent of the PFC command flow, because it has its own name, data structure, and parameter definition style.

**Figure 7:**
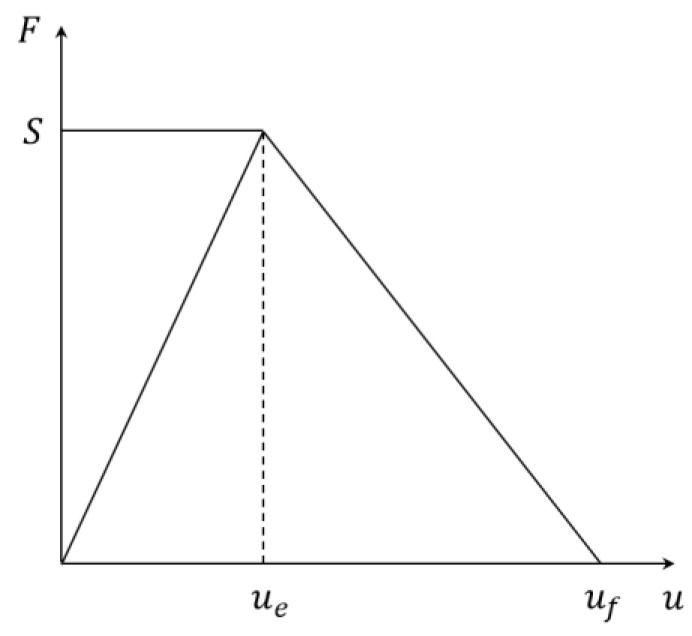
Linear cohesive constitutive model.

Based on a previous researcher’s work [33], a linear cohesion model was established, as shown in Figure 7. Normal and shear stiffness ($k_{n}$, $k_{s}$), normal and shear bond force ($S_{n}$, $S_{s}$), friction coefficient (μ), normal and shear relative displacement ($u_{n}$, $u_{s}$) are all used in the model as control parameters. In addition, the damage parameters in the normal and shear directions are assumed to have the same value in the model. This indicates that there is just one scalar damage parameter ($D_{f}$), which is commonly accepted in quasi-brittle material modeling [34,35,36].

The linear cohesion constitutive model that has been established is as follows:

(3) $${\begin{array}{c} F_{n}=\left(1-D_{f}\right)k_{n}u_{n}\\ F_{s}=\left(1-D_{f}\right)k_{s}u_{s} \end{array}}_{\;}$$

Here, $F_{n}$ is the normal force, $F_{s}$ is the shear force; the equation of $D_{f}$ is:

(4) $$D_{f}=\max(D_{f}^{n},D_{f}^{s})\;=\{\begin{array}{l} 0\\ 1-\frac{u_{e}^{n,s}}{u_{n,s}}\left(\frac{u_{f}^{n,s}-u_{n,s}}{u_{f}^{n,s}-u_{e}^{n,s}}\right) \end{array}\;\;\;\;{\begin{array}{l} u_{n,s}\leq u_{e}^{n,s}\\ u_{n,s}>u_{e}^{n,s} \end{array}}_{\;}$$

where $u_{n,s}$ is the relative normal or shear displacement between two particles, $u_{e}^{n,s}$ is the displacement corresponding to the normal or shear elastic limit, $u_{f}^{n,s}$ is the parameter governing the slope of the normal or shear softening curve.

For in-plane loads, many failure envelopes have been proposed, including linear [37], elliptic [38], and quadratic [39]. An elliptical envelope is employed in this model.

(5) $${\left(\frac{F_{n}}{S_{n}}\right)}^{2}+{\left(\frac{F_{s}}{S_{s}}\right)}^{2}=1_{\;}$$

## 4. Validation of the Linear Cohesion Model

**Figure 8:**
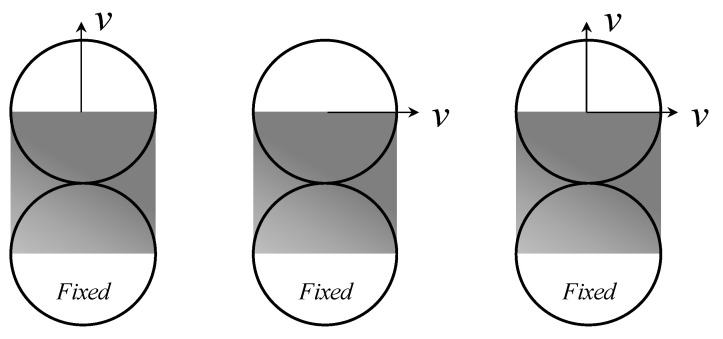
Constitutive model verification.

In the discrete element program PFC5.0, the linear cohesion model created above was implemented as a user-defined model. Two particles were used to check the load–displacement curves under the conditions of Mode-I fracture, Mode-II fracture, and Mixed-mode fracture in order to evaluate the reliability of the constructed model. Figure 8 depicts simulations of the two particles under various loading scenarios. The two particles have a diameter of 0.1 m, and the other microscopic properties are stated in Table 3. Until the contact is broken, one particle remains motionless while the other maintains a steady velocity.

### 4.1. Validation of the Linear Cohesion Model under Mode-I Fracture and Mode-II Fracture

**Figure 9:**
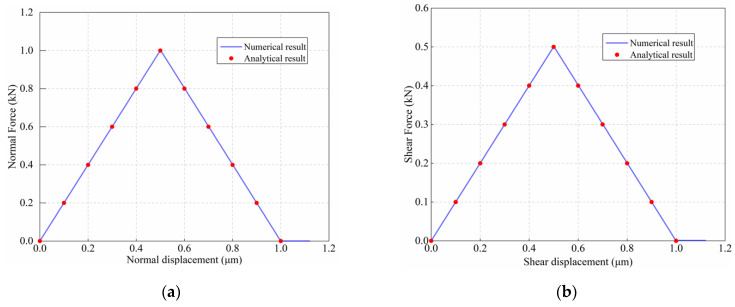
Comparison between analytical and numerical results of force–displacement curves: (**a**) Mode-I fracture; (**b**) Mode-II fracture.

The load displacement curves of two particles under Mode-I fracture and Mode-II fracture can be analytically determined from Equation (3)–(5) first, and then the simulation results obtained by DEM simulation are compared with the analytical results, as shown in Figure 9.

The load–displacement curves obtained from the DEM simulation are in perfect agreement with the analytical results, as shown in Figure 9. Therefore, the developed linear cohesion model is ready for the simulation of Mode-I and Mode-II fractures.

### 4.2. Verification of the Linear Cohesion Model under Mixed-Mode Fracture

**Figure 10:**
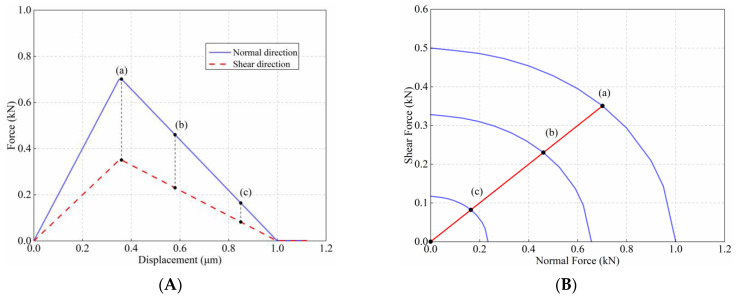
(**A**) Force–displacement relationship in normal and shear direction; (**B**) the evolution of force states as the yield surface shrinks.

The normal and shear behavior during the Mixed-mode loading for the two particles is shown in Figure 10A. Three loading states corresponding to different damage levels are selected from Figure 10A to plot the corresponding yield surfaces, as shown in Figure 10B.

Figure 10A shows that as the normal and shear displacements increase, the normal and shear forces increase first and then decrease linearly, and the two trends are consistent. The three selected points are all precisely located on their corresponding yield surfaces, as shown in Figure 10B. If the conditions of Equation (6) are satisfied while setting the micro-parameters, the yield surfaces under different damage states are parallel.

(6) $$\frac{k_{n}u_{f}^{n}}{S_{n}}=\frac{k_{s}u_{f}^{s}}{S_{s}}\;$$

## 5. Parameter Analysis of Linear Cohesion Model

**Figure 11:**
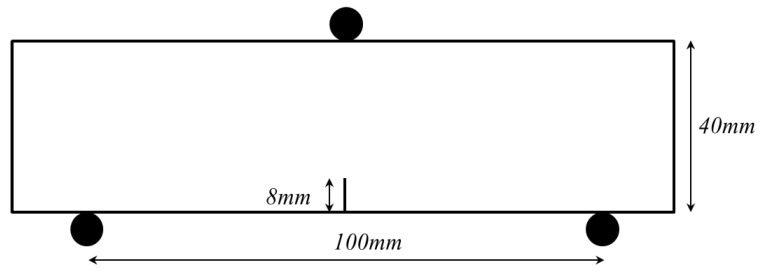
Specimen geometry and boundary condition for the notched mortar beam.

As described in the previous section, the linear cohesion model is controlled by six microscopic parameters, namely, stiffness ($k_{n}$ and $k_{s}$), bond force ($S_{n}$ and $S_{s}$), and softening parameter ($u_{n}$ and $u_{s}$). In this section, the effects of these microscopic parameters on the simulation results of mortar specimens are studied successively. Note that the mortar mixture, as listed in Table 4, is different when compared with the one in Section 2. In this study, when one parameter changes, the other microscopic parameters remain the same. In order to save the time of calibration and simulation, the influence of microscopic parameters is studied by a notched mortar beam with a relatively small size of 40 mm × 40 mm × 160 mm, as shown in Figure 11.

**Figure 12:**
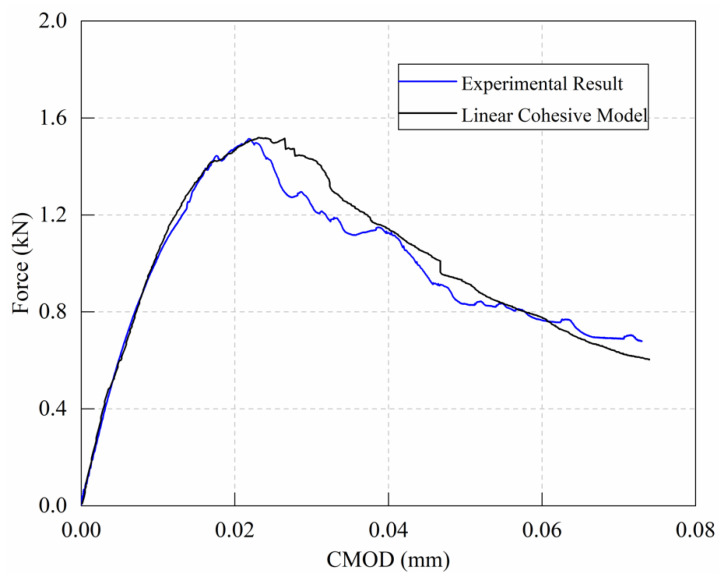
Experimental and simulation results of notched mortar beam.

The micro-parameter calibration process of the notched mortar beam was performed using the method proposed previously by the authors [26]. The obtained parameters are listed in Table 5, and the simulation results are shown in Figure 12.

### 5.1. Effect of Stiffness

**Figure 13:**
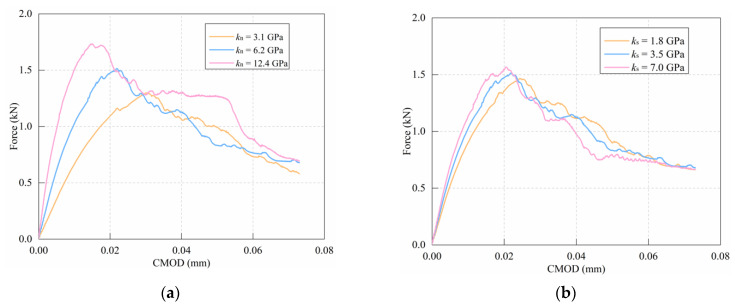
Effect of stiffness: (**a**) normal stiffness; (**b**) shear stiffness.

Three different stiffness values ($0.5k_{n,s}$, $1.0k_{n,s}$, and $2.0k_{n,s}$) were selected to study the effect of normal and shear stiffness on the simulation results of notched mortar beams. The simulation results are shown in Figure 13.

Figure 13a shows that the ascending segment of the curve becomes steeper as the normal stiffness increases. The peak value increases as the normal stiffness ($k_{n}$) increases as well. Figure 13b shows that raising the shear stiffness ($k_{s}$) has the same tendency as increasing the normal stiffness, but with a smaller impact on the peak and slope.

### 5.2. Effect of Bond Force

**Figure 14:**
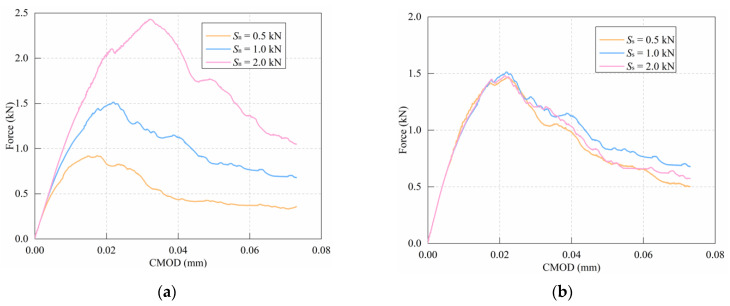
Effect of bond force: (**a**) normal bond force; (**b**) shear bond force.

Three different bond force values ($0.5S_{n,s}$, $1.0S_{n,s}$, and $2.0S_{n,s}$) were selected to study the effect of normal force and shear force on the simulation results. The simulation results of notched mortar beams are shown in Figure 14.

As illustrated in Figure 14a, the peak value tends to rise obviously as the normal bond force ($S_{n}$) rises. However, as shown in Figure 14b, increasing the shear bond force ($S_{s}$) does not appear to have a significant effect on the load–crack opening displacement curve. The reason is that the Mode-I fracture is dominant in the failure process of three-point bending test; hence, the increase in normal bond force is much more effective than the increase in shear bond force.

### 5.3. Effect of Softening Parameters

**Figure 15:**
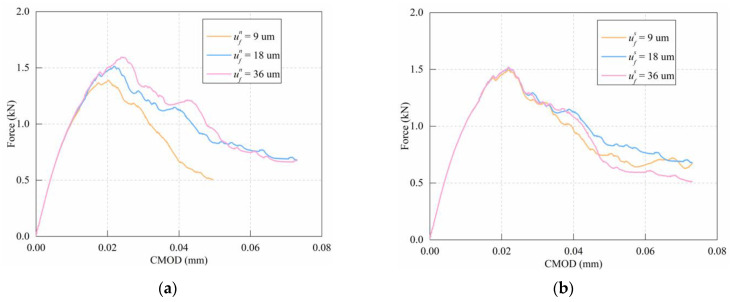
Effect of softening parameter: (**a**) normal softening parameter; (**b**) shear softening parameter.

Three different values of softening parameters ($0.5u_{f}^{n,s}$, $1.0u_{f}^{n,s}$, and $2.0u_{f}^{n,s}$) were selected to study the influence of normal softening parameters and shear softening parameters on the failure behavior of notched mortar beams. The simulation results are shown in Figure 15.

The simulation results show that larger values of normal softening parameter $u_{f}^{n}$ lead to higher values of peak force (Figure 15a). The post-peak softening branch appears to decay more slowly. The reason is that as the normal softening parameter increases, the normal force between the particles that can be obtained according to Equation (1) consequently increases. This will affect the bearing capacity of the beam at the macro level, characterized by a smoother decline of the load–crack opening displacement curve. For comparison, the effect of shear softening parameter $u_{f}^{s}$ on the three-point bending test of the notched mortar beam is insignificant (Figure 15b). This is because in the three-point bending test, the influence of shear partial softening is limited.

## 6. Simulation of Fracture Behavior of the Three-Point Bending Beam by the Linear Cohesion Model

The newly developed linear cohesion model is now applied to the simulation of a three-point bending test on the notched beam specimen with a size of 100 mm × 100 mm × 400 mm, as mentioned in Section 2. The parameter calibration technique and accompanying simulation settings remain the same as discussed before; Table 6 shows the acquired microscopic parameters of the linear cohesion model.

**Figure 16:**
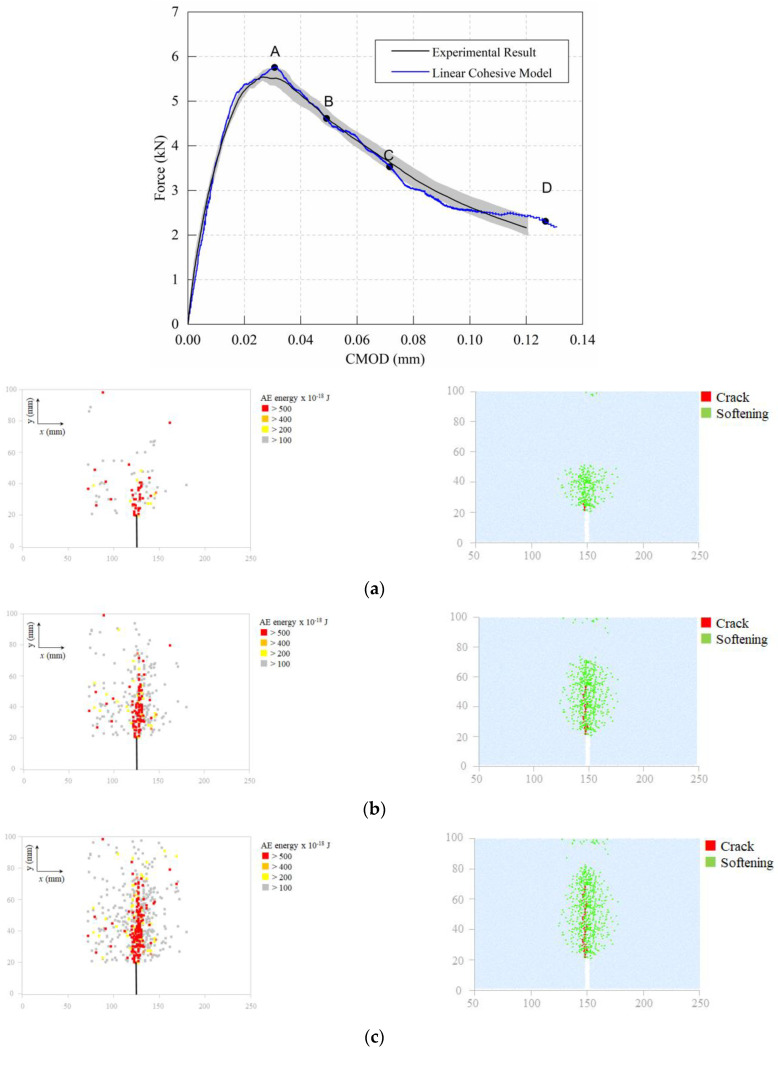

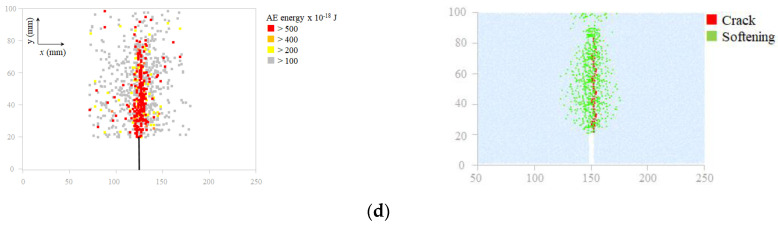
Comparison of AE results and simulation results in the center section of the beam: (**a**) Point A (peak force); (**b**) Point B (post-80% of peak force); (**c**) Point C (post-60% of peak force); (**d**) Point D (post-40% of peak force).

The simulation results of the load–crack opening displacement curve are compared to the experimental results, as shown in Figure 16. One may observe that the load–crack opening displacement curve obtained from the simulation agrees very well with the experimental results. Subsequently, four stages of the fracture process, i.e., 100%, 80%, 60%, and 40% of the peak load, were selected for comparison with the results of acoustic emission (AE) during the experiment from the author’s previous work [40].

Previous studies [41,42] have shown that accumulative levels of acoustic emission can provide relevant information on the fracture zone of mortar and concrete. Therefore, four energy levels are defined by classifying a 2D AE localization map. From the present study depicted in Figure 16, it can be noticed that the high energy level of the acoustic emission event is mainly located in the vicinity of the middle span, which outlines the fracture path in the specimen at different fracture levels. According to the definition from Bažant [3], the tensile stress distribution in the fracture process zone gradually increases from the initial crack tip and reaches tensile strength at the end of the fracture process zone. In the DEM calculation, the simulated cracks are characterized by two groups of contact–failure states: (1) the contact between two adjacent particles enters the displacement softening section (labeled as ‘Softening’ in Figure 16), and (2) the contact–displacement has reached or exceeded the softening parameters, indicating the full failure of contact (labeled as ‘Crack’ in Figure 16). This concept is different from the virtual crack model or the traditional crack model. It can be seen from Figure 15 that when the peak force (point A) is reached, the ‘Crack’ can be found between the particles at the notch tip of the beam. Afterwards, the ‘Crack’ propagates towards the top of the beam, accompanied with an expansion of the ‘Softening’ area; consequently, the bearing force descends progressively (as indicated by points B to D in the figure). The results show that the implemented linear cohesion model successfully simulates the fracture process, and the simulation results are in good agreement with the AE test results.

## 7. Conclusions

In this study, an attempt was made to overcome the limitation of the built-in parallel bond model in the DEM software, for simulating the fracture behavior of quasi-brittle materials.

(1) The built-in parallel bond model was reviewed. The results show that this model can simulate the linear elastic stage and capture the maximum load during the fracture failure of quasi-brittle materials, but it cannot reproduce the nonlinear ascending and the post-peak softening stages. Therefore, the built-in parallel bond model is considered inappropriate for simulating fracture behavior of quasi-brittle materials.
(2) A linear cohesion model for DEM simulation of quasi-brittle materials was constructed, and the reliability of the model verified by investigating the failure behavior of two particles. In addition, the parametric study on the micro-parameters of the newly built linear cohesion model was conducted based on a small-scale notched beam. The results show that the model is able to simulate the fracture behavior of quasi-brittle materials under different failure modes, and the impact of different model parameters are indicated.
(3) The three-point bending test on a notched mortar beam of standard size is simulated using the implemented linear cohesion model. The comparison of experimental and simulation results, with respect to the structural behavior and the fracture process, approves the feasibility of the newly built linear cohesion model.

## Figures and Tables

**Table 1:** Mix design of mortar (kg/m^3^).

Cement	Sand	Water	Superplasticizer	Compressive Strength	Flexural Strength
645	1134	258	7.5	74.5 ± 2.84 MPa	5.8 ± 0.08 MPa

**Table 2:** Micro-parameters determined for the parallel bond model.

Micro-Parameters	Value	Micro-Parameters	Value
Minimum particle diameter [$d_{\min}$] (mm)	0.5	Radius multiplier [λ]	1.0
Ratio of maximum to minimum particle diameter [$d_{\max}/d_{\min}$]	1.5	Ratio of normal to shear bond stiffness [${\bar{k}}_{n}/{\bar{k}}_{s}$]	3.0
Effective modulus [$E_{c}$] (GPa)	31.4	Effective bond modulus [${\bar{E}}_{c}$] (GPa)	31.4
Ratio of normal to shear stiffness [$k_{n}/k_{s}$]	3.0	Moment contribution factor [β]	1.0
Friction angle [ϕ] (°)	26.6	Tensile strength [$\sigma_{c}$] (MPa)	32.7
Cohesion strength [c] (MPa)	32.7		

**Table 3:** Micro-parameters used in the two-particle tests.

Micro-Parameters	Value
Friction coefficient [μ]	0.5
Normal stiffness [$k_{n}$] (GPa)	2.0
Shear stiffness [$k_{s}$] (GPa)	1.0
Normal bond force [$S_{s}$] (kN)	1.0
Shear bond force [$S_{s}$] (kN)	0.5
Normal softening parameter [$u_{f}^{n}$] (μm)	1.0
Shear softening parameter [$u_{f}^{s}$] (μm)	1.0

**Table 4:** Mix design of mortar in the test for parameter analysis of the linear cohesion model (kg/m^3^).

Cement	Sand	Water	Superplasticizer	Compressive Strength	Flexural Strength
668	1175	301	0	54 MPa	4.92 MPa

**Table 5:** Micro-parameters of the linear cohesive model obtained after calibration for small beam test.

Micro-Parameters	Value	Micro-Parameters	Value
Minimum particle diameter [$d_{\min}$] (mm)	0.5	Normal stiffness [$k_{n}$] (GPa)	6.2
Ratio of maximum to minimum particle diameter [$d_{\max}/d_{\min}$]	1.5	Ratio of normal to shear bond stiffness [$k_{n}/k_{s}$]	1.77
Normal bond force [$S_{n}$] (kN)	1.0	Shear bond force [$S_{s}$] (kN)	1.0
Normal softening parameter [$u_{f}^{n}$] (μm)	18	Shear softening parameter [$u_{f}^{s}$] (μm)	18
Friction coefficient [μ]	0.5		

**Table 6:** Micro-parameters of linear cohesive model applied in the structural simulation.

Micro-Parameters	Value	Micro-Parameters	Value
Minimum particle diameter [$d_{\min}$] (mm)	0.5	Normal stiffness [$k_{n}$] (GPa)	24.5
Ratio of maximum to minimum particle diameter [$d_{\max}/d_{\min}$]	1.5	Ratio of normal to shear bond stiffness [$k_{n}/k_{s}$]	1.77
Normal bond force [$S_{n}$] (kN)	1.8	Shear bond force [$S_{s}$] (kN)	1.8
Normal softening parameter [$u_{f}^{n}$] (μm)	35	Shear softening parameter [$u_{f}^{s}$] (μm)	35
Friction coefficient [μ]	0.5		

## Data Availability

Data are available on request due to restrictions, e.g., privacy or ethical.

## Nomenclature

$k_{n}$	Normal stiffness
$k_{s}$	Shear stiffness
$E_{c}$	Young’s modulus of mortar
${\bar{k}}_{n}$	Bond normal stiffness
${\bar{k}}_{s}$	Bond shear stiffness
c	Cohesion strength
$\sigma_{c}$	Tensile strength
${\bar{E}}_{c}$	Effective modulus
γ	Maximum shear strength multiplier
$\bar{R}$	Bond radius
β	Moment contribution factor
ϕ	Friction angle
$R_{1}$	Radius of the bonded particle 1
$R_{2}$	Radius of the bonded particle 2
$F_{n}$	Normal force	
$F_{s}$	Shear force	
$\sigma_{n}$	Normal contact stress	
$\tau_{\max}$	Maximum shear stress	
$\tau_{s}$	Shear contact stress	
μ	Friction coefficient	
$S_{n}$	Normal bond force	
$S_{s}$	Shear bond force	
$u_{f}^{n}$	Normal softening parameter	
$u_{f}^{s}$	Shear softening parameter	
$u_{n}$	Relative normal displacement	
$u_{s}$	Relative shear displacement	
$u_{e}^{n}$	Displacement corresponding to the normal elastic limit	
$u_{e}^{s}$	Displacement corresponding to the shear elastic limit	
